# Mapping the role of sexuality in adolescent mental health and substance use

**DOI:** 10.1098/rsos.230955

**Published:** 2024-06-26

**Authors:** Jack L. Andrews, Duncan E. Astle, Jonathan S. Jones, Sarah-Jayne Blakemore

**Affiliations:** ^1^ Department of Experimental Psychology, University of Oxford, Oxford, UK; ^2^ University College, University of Oxford, Oxford, UK; ^3^ MRC Cognition and Brain Sciences Unit, University of Cambridge, Cambridge, UK; ^4^ Department of Psychiatry, University of Cambridge, Cambridge, UK; ^5^ Department of Psychology, University of Cambridge, Cambridge, UK

**Keywords:** depression, sexual minority, network analysis, substance use

## Abstract

Individuals who belong to a sexual minority are at greater risk of adverse health and social outcomes. These effects are observed during adolescence when many mental health problems, such as depression, first emerge. Here, we used a network analytic approach to better understand the role that sexual minority status plays in the association between depression, interpersonal difficulties and substance use in a large sample of mid-adolescents. In doing so, we used data from 8017 fourteen year olds from the UK’s Millennium Cohort Study, of which 490 self-identified as belonging to a sexual minority. We found that sexual minority status was highly central in the network and connected to multiple adverse outcomes, sometimes directly and sometimes indirectly. The largest single association was between sexual minority status and depression, and this link mediated multiple negative associations with being in a sexual minority. The shortest path to drinking, poor social support and closeness with parents and victimization occurred via depression. The shortest path to smoking and drug use occurred via conduct problems. We also identified three distinct profiles of adverse outcomes among those belonging to a sexual minority, highlighting the heterogeneous nature of this group.

## 1. Introduction

Adolescence, defined as the period of life between 10 and 24 years [1], is a time of heightened risk for the onset of mental health problems, with approximately 75% of all adult mental health problems first appearing before the age of 24 years [2]. Adolescents who identify as belonging to a sexual minority (for example, identifying as gay, lesbian and bisexual) are at greater risk of developing mental health problems, such as depression, relative to their heterosexual peers [3,4]. Adolescent depression rarely occurs in isolation but instead has reciprocal relationships with a number of health risk behaviours such as smoking and drinking [5,6]. Relative to heterosexual adolescents, sexual minority adolescents are also at a greater risk of engaging in a number of these adverse health risk behaviours and of other negative outcomes such as being the targets of peer victimization [7–9].

There is therefore a need for a deeper understanding of the co-occurring nature of these adverse outcomes in sexual minority young people [10]. What is currently unknown is whether these negative outcomes are directly associated with sexual minority status, or whether they are a downstream consequence of other negative outcomes. For example, are increased rates of substance use directly associated with sexual minority status? Or are they a consequence of increased victimization, which results from belonging to a sexual minority? This kind of information is vital for tailoring support for this community.

It has been widely hypothesized that sexual minority adolescents are at increased risk of developing depression because of minority stress, which is sustained through the experience of stigma and homophobic-related victimization [11,12]. One study found that ‘feeling like a burden’ to others was an important mechanism explaining higher levels of depression among sexual minority young people. This perceived burdensomeness also mediated the association between sexual orientation-related victimization and depression [13]. The authors attribute this perceived burdensomeness to a lack of belongingness—a feeling of social safety associated with belonging to a social group [13]. This lack of belonging is perhaps especially salient among sexual minority young people, given that adolescence is a time of life where belonging is particularly important and can contribute to mental well-being [14]. However, it is important to note that much of the work on minority stress has been understood within the context of Western samples. For example, one study found only partial support for role of minority stress in understanding inequalities in mental health among sexual minority men in China [15]. For example, the authors found that concerns around hiding one’s orientation did not directly predict psychological distress, which counters much evidence from the West. They explain this contradictory finding within the context of a strong desire to conform to societal norms, which may outweigh possible stress attributed to revealing their sexual orientation [15].

Sexual minority youth are often faced with the worry of family acceptance following disclosure of their sexual orientation [16]. Stress attributed to ‘coming out’ is unique to sexual and gender minority young people and one way in which minority-related stress is likely to impact mental health outcomes. One study found that sexual minority youth in the USA were more likely to experience verbal and physical abuse by family members following disclosure of their orientation [17]. This is likely to have a significant impact on these young people’s mental health, given that abuse from family members is a known risk factor for poor lifetime mental health [18]. In addition, individuals who fear rejection from family and peers are more likely to report higher levels of depression, compared with those who do not [19]. However, sexual minority adolescents whose parents support them are more likely to report increased levels of self-esteem and reduced levels of depression, compared with those who do not have family support [20]. Having a supportive family may also buffer against negative experiences outside of the home, such as bullying, and this might lead to higher levels of resilience [21].

In the first decade of this century, the average age of ‘coming out’ was approximately 14 years (in the USA [22]), which is lower than the previous decade, when it was around 16 years of age [23]. This age coincides with a period of development during which peer influence and sensitivity to social rejection is heightened [24–27]. Adolescents who report being frequently victimized by peers at the age of 13 have a substantially higher likelihood of developing depression and anxiety at the age of 18, compared with non-victimized peers [28,29]. These effects can be long-lasting and a number of studies have shown a strong link between victimization during adolescence and later risk for poor mental health [30–32]. Sexual minority adolescents report increased rates of victimization relative to their heterosexual peers [7], and it has been shown that sexual minority-specific victimization mediates the relationship between sexual minority status and depressive symptoms, as well as suicidality [33].

In addition, adolescents who are victimized are at a higher risk for substance use, such as binge drinking, smoking or drug use [34,35]. Work examining this association within the context of sexual minority adolescents has shown that anti-lesbian, gay, bisexual and trans victimization is associated with greater affiliation to ‘deviant’ peer groups (e.g. engagement with antisocial behaviours such as stealing), which is in turn associated with greater substance use [36]. Interestingly, longitudinal work among sexual minority males has shown that the association between victimization, mental health and substance use decreases between adolescence and adulthood, which is possibly owing to reductions in victimization experiences [37]. Collectively, these findings demonstrate the complex, and indirect nature of the associations between sexual minority status and substance use and mental health outcomes, pointing to the importance of peer relationships and victimization experiences in precipitating adverse experiences.

Given this, peer relationships are also likely to be a predictor of positive outcomes for sexual minority young people. This is evidenced from data showing that sexual minority youth who report retaining friends following disclosure of their orientation also report higher levels of self-esteem and lower levels of depression, compared with those who lost friends owing to disclosing their orientation [38]. Furthermore, sexual minority youth who have other sexual minority friends are less likely to report depression and to feel the negative effects of victimization, compared with those who do not [39]. The formation of friendships among sexual minority adolescents might thus buffer against the negative effects of peer victimization by increasing a sense of belonging and security and by experiencing and empathizing about a shared stressor [39].

In order to further understand why sexual minority status appears to confer particular risk for such a wide number of negative outcomes, here we build a network model in which we explore the role that sexual minority status plays in connecting three broad domains of interest—depression, interpersonal relationships and health risk behaviours—in a large sample of British mid-adolescents who are part of an ongoing longitudinal birth cohort study (Millennium Cohort Study (MCS)). Previous work using this dataset has shown that young people who report same sex attraction are at a greater risk of experiencing poor outcomes in each of these three domains [7]. Given that these variables are likely to have complex interactions with each other, network analysis presents an appealing method with which to explore co-dependences and potential mediating factors between such variables [40,41]. In addition, network analysis is an attractive method as it examines partial correlations between each node, or variable, in the network, while controlling for all other variables in the network. We hypothesized that some adverse associations with minority status will be direct, and others will be downstream consequences of other more indirect associations. Our network analysis allows us to distinguish these two types of relationships.

## 2. Methods

### 2.1. Sample

The MCS is a UK cohort study following children born between 2000 and 2002. There were 19 519 children initially recruited. In the current study, we make use of data from the sixth sweep (January 2015–March 2016), when cohort members were 14 years old. The total sample size of cohort members followed up at this time point was 11 884. The two primary reasons for the reduced response rate at wave 6 were non-issued questionnaires where participation was not invited given absence at two prior waves (19.9%) and refusal (15.7%). The MCS study received ethical approval from the National Research Ethics Service Committee London—central (reference 12/LO/1786). Inclusion in the analysis was dependent on complete data across all variables included (total *n* = 8017; male = 4078; female = 3939). MCS data are freely available to researchers via the UK Data service (SN: 8156). UK National Health Service Research Ethics approved the MCS and informed consent was obtained from cohort members and their parents.

### 2.2. Measures

#### 2.2.1. Sexuality

Cohort members at the age of 14 years were asked to report if they had (i) ever been attracted to a female, or (ii) a male. Using this measure, we identified sexual minority individuals. This method of determining sexual minority status within this sample has previously been used [7]. It is important to note that the phrasing of this question was not of our choosing, and information about other relevant gender or sexual minorities was not obtained (e.g. individuals who identify as non-binary).

#### 2.2.2. Depressive symptoms

Depressive symptoms were measured using the Child Self-Report version of the short Mood and Feelings Questionnaire (MFQ) [42]. The short MFQ is a valid and reliable measure of depressive symptoms among adolescents [43], and has excellent internal consistency in our sample (Omega = 0.94).

#### 2.2.3. Social support

Social support was measured with three items pertaining to relationships with friends, family and community members, closeness with parents was measured by averaging across two measures relating to closeness with the cohort members mother and father, and victimization was measured across three domains: siblings, other children and cyber bullying.

#### 2.2.4. Conduct problems and peer problems

Conduct problems and peer problems were measured using the parent report subscales of the Strengths and Difficulties Questionnaire (SDQ) [44]. The SDQ and its subscales have shown good internal and external reliability and validity in adolescent samples [45,46].

#### 2.2.5. Smoking and drinking

Smoking and drinking were each assessed using a single item and drug use was assessed using a combination of two items relating to cannabis use and other illegal drug use.

Across all measures, higher scores indicate poorer outcomes and for our measure of sexual minority status, a score of 1 indicates sexual minority status and 0, heterosexual status. See the electronic supplementary material for a more detailed explanation of all the variables included in the analysis.

### 2.3. Statistical analysis

All analyses were conducted in R (version 3.6.2), except for sub-group analyses which employed the Brain Connectivity Toolbox in Python [47]. First, *t*-tests (Bonferroni corrected for multiple comparisons) were conducted to test for differences between sexual minority and heterosexual adolescents on each variable.

A mixed graphical model was estimated on all participants using the ‘mgm’ package (version 1.2-10 [48]), which allows for network estimation when there is a mix of continuous and binary variables. A least absolute shrinkage and selection operator [49] was used to regularize the network. The degree of regularization is set by the extended Bayesian information criteria (EBIC) hyperparameter (between 0 and 0.5). Setting the parameter at 0 errs on the side of discovery, while setting it to 0.5 provides a more conservative estimation—reducing spurious edges but increasing the probability of missing some true edges [50]. The choice of 0.3 was also based on previous simulation work showing that results differ only slightly when the hyperparameter is varied between 0.25 and 0.5. However, this simulation work also highlighted that setting the hyperparameter at 0.5 does not always lead to the true model given an increased chance that true edges are omitted from the model [50,51]. Therefore, we set our EBIC hyperparameter to 0.3, which takes a middle ground between exploration and parsimony when identifying edges in the network, and has been employed in recent studies of a similar nature [52].

The *qgraph* package (version 1.5.4) was used to visualize the network, and the Fruchterman–Reingold algorithm [53] was selected, whereby the most highly connected nodes (corresponding to each variable) are placed more centrally in the network. Edges between nodes represent the association between two variables. Edge thickness and saturation are indicated by the strength of the associations. Green edges represent positive associations while red edges represent negative associations [54]. The presence of node clusters within each network was explored using a spinglass algorithm computed using the *igraph* package (version 1.24). This algorithm identifies communities of nodes with a high proportion of edges within the community and few outside it.

We further estimated node predictability, which is determined by the degree to which each node is predicted by neighbouring nodes in the network (i.e. those it shares a direct edge with). The proportion of variance explained (*r* ^2^) was computed for continuous variables and correct classification (CC) was computed for binary variables [48]. We used the ‘bootnet’ package (version 1.3.0) in order to run a non-parametric bootstrap, in which 1000 permutations were specified, in order to determine the 95% confidence intervals (CIs) for each edge in the network. In order to compare edge weights, we used the bootstrap difference test (significance set at *p* < 0.05). We determined the shortest paths between sexual minority status and all other variables in the network using Dikstra’s algorithm. The shortest path analysis determines the fastest route through the network between two nodes (the strength of each edge weight is accounted for). This process highlights potential pathways that are mediated by additional nodes in the network. We can therefore characterize direct and indirect relationships between nodes. For example, if the shortest path from node A to C was via node B, this would be an indirect relationship. This allows us to model whether certain poor outcomes are directly related to sexual minority status, or whether they are antecedents or consequences of other variables.

We computed a number of centrality measures for each node in the network. These centrality measures indicate the relative importance of a node in maintaining the network composition. Four centrality measures were computed: strength, expected influence, betweenness and closeness. Strength centrality refers to the sum of the strength of edges connected to any one node. Expected influence centrality is similar to strength centrality but does not take the absolute value of edges before summing, therefore providing a measure of overall positive connectivity, in networks with both positive and negative edges [55]. Betweenness centrality refers to the number of times any one node lies on the shortest path between two other nodes, and closeness centrality is the mean distance of a node from all other nodes in the network. Collectively, centrality indices contribute to our understanding of how each node (i.e. variable in our case) interacts within our network by providing us with information regarding the degree to which each node influences network dynamics (i.e. facilitating connectivity), network structure (i.e. the importance of each node in maintaining the composition of the network) and how these contribute to clustering of nodes (i.e. by aiding the detection of communities of variables).

Finally, we assessed whether there was any evidence for different sub-groups of individuals with a sexual minority status using community detection. Scores on all the measures were first correlated between every pair of individuals to create a similarity matrix. To find clusters of highly related individuals, the community Louvain algorithm was applied to the similarity matrix to find the optimal number and assignment of communities to maximize modularity (*Q*), which is the strength of within community associations relative to random chance [56]. To account for the random initiation in the algorithm, a consensus partition was determined across 1000 iterations of the community detection [57]. These analyses were conducted using the Brain Connectivity Toolbox [47] for Python (https://github.com/aestrivex/bctpy).

## 3. Results

### 3.1. Differences in outcomes for sexual minority and heterosexual adolescents

**Figure 1.**
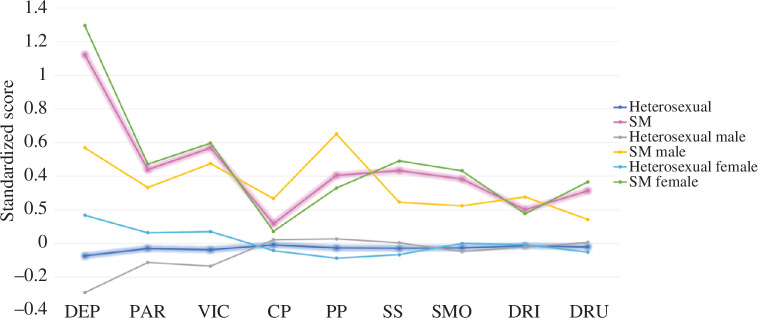
Profile plot of mean standardized *z-*scores of each variable by group (sexual minority (SM) status) in males and females. The SM and heterosexual groups are represented with bold lines, and gender sub-groups by the thin lines. Lines connect scores on each variable between each group and are included for ease of visual comparison. DEP, depression; PAR, closeness to parents; VIC, victimization; CP, conduct problems; PP, peer problems; SS, social support; SMO, smoking; DRI, drinking; DRU, drug use.

A total of 490 cohort members reported same sex attraction and were included within the sexual minority group (males = 116; females = 374). The other 7527 cohort members included in this analysis did not report same sex attraction and were classified as heterosexual (males = 3962; females = 3565). Bonferroni corrected *t*-tests revealed that sexual minority adolescents had significantly poorer outcomes in all variables assessed (corrected for nine comparisons; *p* < 0.05), except for conduct problems, which did not survive correction for multiple comparisons (uncorrected *p-*value = 0.007). See table 1 for the results of each difference test conducted and the electronic supplementary material for the variables used to assess each outcome. Because of the asymmetric gender split, we repeated this analysis for each gender. Sexual minority and heterosexual females showed significant differences across all variables except conduct problems, while sexual minority and heterosexual males showed significant differences across all variables except conduct problems, drinking, drug use, smoking and social support (see figure 1 for a profile plot of mean standardized *z-*scores for each group and the electronic supplementary material, tables S1 and S2 for the results of each difference test conducted for males and females).

**Table 1.** Results from *t*-tests conducted on each variable of interest between sexual minority and heterosexual adolescents (adjusted *p-*value = Bonferroni corrected for multiple comparisons).

	sexual minority (SM) (*n* = 490)		heterosexual (*n* = 7527)		difference test	
variable	mean	s.d.	mean	s.d.	*t*‐test	adjusted *p*-value
depression (DEP)	12.21	7.42	5.22	5.45	−20.48	<0.00001
conduct problems (CP)	1.50	1.71	1.30	1.52	−2.44	>0.05
peer problems (PP)	2.24	2.03	1.50	1.68	−7.89	<0.00001
social support (SS)	0.88	1.19	0.47	0.87	−7.57	<0.00001
victimization (VIC)	7.98	3.53	6.14	2.97	−11.23	<0.00001
closeness to parents (PAR)	4.56	1.55	3.85	1.48	−9.71	<0.00001
smoking (SMO)	1.60	1.19	1.25	0.78	−6.11	<0.00001
drinking (DRI)	1.46	0.67	1.31	0.76	−4.15	<0.00001
drugs (DRU)	0.13	0.38	0.05	0.23	−4.80	<0.00001

### 3.2. Network analysis

#### 3.2.1. Network estimation and visualization

**Figure 2.**
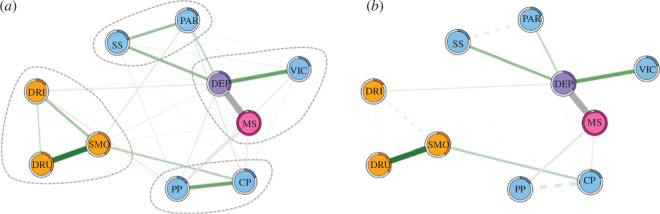
(*a*) Network diagram. Yellow nodes represent substance use variables, blue represent interpersonal difficulties, purple correspond to depression and pink correspond to sexual minority status. Dashed lines represent the communities of nodes detected from the spinglass algorithm. Positive relationships between nodes are represented in green, negative in red. Relationships between categorical variables are grey. (*b*) Shortest paths from sexual minority status. The shortest path between sexual minority status and depression, peer problems and conduct problems shows that a direct route is the dominant pathway. The shortest path to smoking and drug use occurred via a mediated route through conduct problems and the shortest path to social support, closeness with parents and victimization occurred via depression. Dashed lines represent associations that do not lie on the shortest path. DEP, depression; MS, minority status; VIC, victimization; SS, social support; PAR, closeness to parents; PP, peer problems; CP, conduct problems; DRU, drug use; SMO, smoking; DRI, drinking.

The estimated network comprising all 10 variables is shown in figure 2*a*. Four communities of variables were detected using the spinglass algorithm: (i) sexual minority status, depression and victimization; (ii) social support and closeness to parents; (iii) peer problems and conduct problems; and (iv) drinking, smoking and drug use (highlighted in figure 2). Exploratory analyses showed that the structure of the edges in the network was the same for males and females. However, females showed greater connectivity within the network, relative to males (see the electronic supplementary material for details).

#### 3.2.2. Node predictability

Sexual minority status had a CC score of 0.94. This suggests that approximately 94% of the time, individuals are correctly classified as either heterosexual or sexual minority given their scores on all variables that share a direct edge with sexual minority status. Depression had the highest *r* ^2^ value of all nodes in the network (*r* ^2^ = 0.34) and drinking had the lowest (*r* ^2^ = 0.14; see the electronic supplementary material, table S3 for the predictability values of all nodes).

#### 3.2.3. Edge weight differences

Sexual minority status showed the largest unique relationship with depression (edge weight = 0.51, 95% CI = [0.51, 0.60]). In addition to depression, sexual minority status also shared direct edges with peer problems (edge weight = 0.18, 95% CI = 1.51, 0.06]), conduct problems (edge weight = −0.11, 95% CI = [−6.50, 0.07]), smoking (edge weight = 0.07, 95% CI = [4.62, 0.04]) and victimization (edge weight = 0.05, 95% CI = [3.39, 0.04]). The edge between sexual minority status and depression was significantly larger than the edges between sexual minority status and all other variables (*p* < 0.05). None of the other edges with sexual minority status was significantly different in size (*p* > 0.05; electronic supplementary material, table S4). Bootstrapped 95% CIs are presented in the electronic supplementary material, figure S1.

#### 3.2.4. Shortest path analyses

The absence of direct edges between sexual minority status and drinking, drug use, closeness with parents and social support demonstrates indirect relationships between sexual minority status and these variables, occurring through other variables in the network (notably depression). The shortest path between sexual minority status and depression, peer problems and conduct problems shows that a direct route is the dominant pathway. The shortest path to smoking and drug use occurred via a mediated route through conduct problems and the shortest path to social support, closeness with parents and victimization occurred via depression (figure 2*b*).

#### 3.2.5. Centrality measures

**Figure 3.**
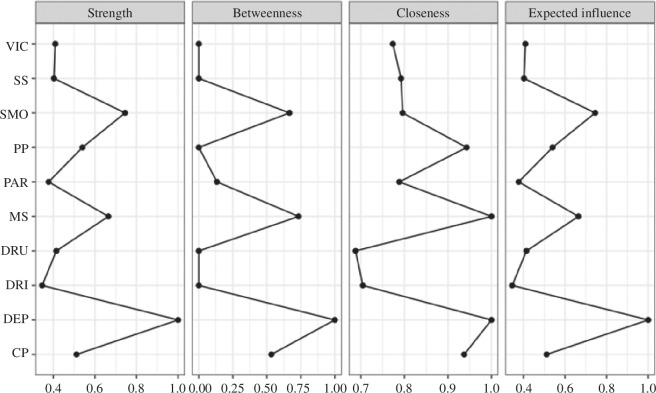
The relative centrality scores for strength, betweenness, closeness and expected influence are plotted for each node within each network. Symptom nodes are represented along the *y*-axis and the relative centrality scores are represented on the *x*-axis, from 0 to 1 (standardized). DEP, depression; MS, minority status; VIC, victimization; SS, social support; PAR, closeness to parents; PP, peer problems; CP, conduct problems; DRU, drug use; SMO, smoking; DRI, drinking.

Centrality scores were computed for each node in the network (see figure 3). Depression had the highest strength and expected influence centrality, followed by smoking and sexual minority status. Depression had the highest betweenness centrality, followed by minority status and then smoking. Depression and sexual minority status shared the highest closeness centrality, followed by peer problems. The stability of these centrality measures is displayed in the electronic supplementary material, figure S2.

### 3.3. Sexual minority status sub-groups

**Figure 4.**
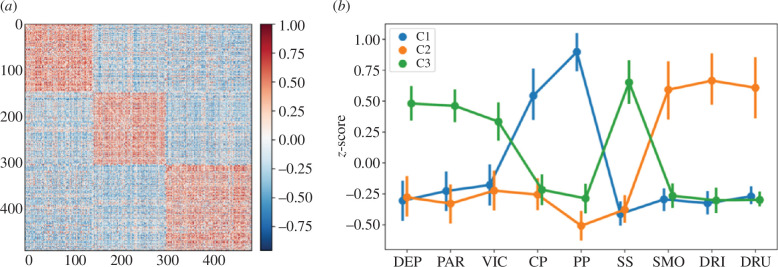
Clustering in individuals with a sexual minority status. Consensus community detection on Pearson correlations between individuals revealed three subgroups of highly related individuals (*Q* = 0.42) over 1000 iterations (*a*). The subgroups had distinct profiles on *z*-standardized scores (*b*) where error bars denote 95% CIs from 1000 bootstrapped resamples. C1 (*n* = 148; 30%), C2 (*n* = 157; 32%) and C3 (*n* = 185; 38%). DEP, depression; VIC, victimization; SS, social support; PAR, closeness to parents; PP, peer problems; CP, conduct problems; DRU, drug use; SMO, smoking; DRI, drinking.

Consensus community detection revealed three distinct clusters of individuals with a sexual minority status (*Q* = 0.42): C1 (*n* = 148), C2 (*n* = 157) and C3 (*n* = 185). Individuals were highly related within each cluster and each cluster showed a distinct profile (figure 4). Group differences were assessed with Bonferroni-corrected one-way ANOVAs and pairwise *t*-tests (see the electronic supplementary material, table S5). Overall, C1 had the highest peer and conduct problems; C2 had the highest smoking, drinking and drug use; and C3 had the highest depression and victimization, and the poorest parent and social support. This shows that not all young people belonging to a sexual minority experience the same negative outcomes, but rather they fall into distinct groups characterized by unique profiles of adverse experiences.

## 4. Discussion

We set out to explore the complex links between sexual minority status and depression, interpersonal difficulties and substance use in a large sample of 14-year-old adolescents. We first replicated, using a different analytic method, a previously reported effect in this sample [7], showing that across nearly all variables included, sexual minority adolescents had poorer outcomes than their heterosexual peers. We then extended these findings by employing network analysis. This revealed that sexual minority status sits at centre of a network of adverse outcomes, with both direct and indirect relationships with multiple negative associations.

Adolescents who belong to a sexual minority are likely to experience depression and victimization at much higher rates than their heterosexual counterparts [3,4,7,9]. In our network model, sexual minority status formed a cluster with depression and victimization. This shows that these variables were uniquely related to one another, when controlling for all other variables in the network. The implications are that the scores on any one of these variables are more likely to influence the scores of variables within their community than those outside of their community. Therefore, associations between nodes within each community are assumed to be stronger than with nodes outside the community. Other communities of nodes that were identified included those relating to substance use (smoking, drinking and drugs), social and parental support and relationships with peers (peer problems and conduct problems).

Overall, sexual minority status and depression had the highest centrality scores in the network, suggesting their importance in maintaining the structure and connectivity of the network. For example, sexual minority status and depression had the highest betweenness centrality indicating their important role in connecting otherwise disparate variables in the network. That is, these two variables are probably mediators between other adverse outcomes in the network. Sexual minority status and depression also held the highest closeness centrality, indicating that these two nodes are the most closely connected to all others in the network. Therefore, changes to these variables with high centrality are more likely to impact other variables in the network, and vice versa, relative to less central variables [58]. Indeed, the single largest predictor of depression was sexual minority status. Our finding that depression had the highest predictability score of all continuous measures suggests its controllability is highly influenced by neighbouring nodes in the network. That is, interventions to target adjacent nodes (e.g. social support and victimization) are likely to have a reasonable degree of control, or influence, on depressive symptoms [59]. In addition, the very high CC score (94%) observed for sexual minority status underlines the impact adjacent nodes have on sexual minority young people. Combined with findings that sexual minority young people are at a significantly inflated risk of experiencing depression, peer problems and victimization, the fact that these nodes also shared a direct edge with minority status highlights their potential as important intervention targets.

It is also worth noting that, while our network model is undirected and the model assumes that relationships are bidirectional, given the nature of the variables included here, we can reasonably assume the direction of effect is from sexual minority status towards depression. This relationship was significantly stronger than the relationships between all other variables and sexual minority status. This is striking when we consider the other variables included in the network (e.g. victimization and substance use) and how regularly they are associated with poor mental health. However, it is important to note that we cannot make causal claims about the associations within our cross-sectional network. While the associations we observe do account for the other nodes in the network, we cannot rule out the possibility that additional, unmeasured, factors (e.g. school climate) influence these findings. Our approach highlights potential causal pathways through which poor outcomes may materialize for sexual minority young people, but these need to be formally tested with causal inference methods.

Our analyses provide further insight into why sexual minority adolescents show such wide-ranging poor outcomes. While some of the relationships between sexual minority status and other variables in our network were direct (e.g. depression, peer and conduct problems), several observed relationships were indirect. For example, victimization, poor closeness with parents, poor social support and drinking behaviour were not directly associated with sexual minority status but were indirectly related via depression (which held the strongest relationship with sexual minority status in the network). That is, poor outcomes in these domains for sexual minority adolescents can be understood as antecedents or consequences of depression. There is an extensive literature showing that depression during adolescence is bidirectionally related to excessive drinking, poor family relationships and poor social support [60–62]. The fact that adolescents belonging to a sexual minority, relative to heterosexual adolescents, reported higher scores on each of these variables, may therefore be both a consequence and cause of the increase in incidence of depression among this group of young people.

We also show that drug use and smoking were indirectly related to sexual minority status, representing antecedents or consequences of conduct problems, identified using shortest path analysis. This suggests that variance within this measure is likely to be mediating the association between sexual minority status and substance use. Given that sexual minority adolescents are more likely to report increased rates of health risk behaviours compared with their heterosexual counterparts, it is possible that interventions aimed at improving peer relationships and conduct problems—which formed a cluster of related variables in our network—among sexual minority adolescents might have positive effects in reducing engagement in certain health-risk behaviours [7,9]. It is important to note that engagement in risky health behaviours may also be a product of ‘adolescent-onset’ conduct problems, which are known to be less stable over time (i.e. behavioural problems are likely to be of a less aggressive nature and more likely to desist by adulthood), reflecting a possible increased desire to conform to unhealthy peer norms and subsequent engagement in risky behaviours [63,64].

We identified three distinct subgroups of sexual minority adolescents, each with a unique profile of adverse outcomes that mirrored the clusters of nodes in the network diagram. Reaffirming the strong association between sexual minority status and depression; depression and victimization were elevated in all subgroups relative to heterosexual adolescents. Furthermore, sexual minority adolescents who reported poor parental and social support (C3) felt the most depressed and victimized. This converges with the strong, direct links between depression and victimization, parental support and social support observed in the network diagram, and suggests that parental and social support may be important protective factors against increasingly higher levels of depression in sexual minority adolescents [65]. Our findings also suggested distinct subgroups with peer and conduct problems (C1) versus those who more regularly smoke, drink and use drugs (C2). This suggests that only some sexual minority adolescents participated in risk-taking behaviours and, interestingly, that reported peer and conduct problems were relatively distinct from self-reported social support.

Several interacting factors are likely to explain this observed heterogeneity. One factor that was not studied here was the extent to which one’s peers also reported high scores on each variable. We know that adolescence is a time of heightened peer influence [26,66] and, therefore, the degree to which one’s peers engage in certain behaviours may be a driving force for one’s own engagement. For example, in our data, we observed one community of sexual minority young people who engaged in substance use but reported low scores on all other variables, suggesting their engagement in substance use was not strongly related to high levels of depression or victimization. Therefore, an outstanding question is whether individual differences in susceptibility to peer influence among sexual minority young people can explain these heterogeneous outcomes. Future work should seek to examine the role of peer influence on outcomes among sexual minority adolescents, given the salience of the peer environment during this stage of life. Overall, these findings highlight that the experiences of sexual minority young people are not homogenous, but rather represent a diversity of difficulties, which may benefit from tailored intervention and management. Additionally, while sexual minority adolescents are on average at greater risk of experiencing these negative outcomes, not all will experience high levels of difficulties. As previous literature suggests, supportive school [67], home [68] and peer environments [38] are strong predictors of positive outcomes among this population.

### 4.1. Limitations

The results presented here should be considered within the context of a number of limitations. First, our measure of sexual minority status is solely based on same sex attraction. The measures within the MCS data did not ask more nuanced questions about sexual attraction, or gender identity, which would be highly relevant. Therefore, the conclusions drawn in the current study are limited to the experience of young people reporting same-sex attraction, and does not account for other potentially intersecting experiences, such as those who identify as non-binary. Future work should seek to co-produce questions of this nature in order to most appropriately represent a diversity of experiences. Second, although some work has shown the age of 14 years to be the average age at which an individual ‘comes out’ as non-heterosexual [22], many young people have not come out to themselves or others by this age. This is particularly marked for males. In addition, it is possible that the high rates of refusal to participate in wave 6 may have led to less representative responses regarding sexual attraction. In short, we are probably underestimating the sexual minority population. Third, the importance of the contribution of interpersonal relationships to depressive symptoms might also differ depending on whether this information is disclosed or not to family and peers, a question that cannot be addressed with this dataset. Fourth, our analysis is entirely based on self-report data from the MCS, which did not collect any qualitative data on this topic. Future work should seek to collect both quantitative and qualitative data to triangulate findings and gain a deeper understanding of the experiences that drive adverse outcomes among sexual minority young people.

Finally, around the time of data collection, marriage equality legalization was being debated in the UK. Subsequent changes in public perceptions and stigma may alter the inter-relationships within the network, weakening links between sexual minority status and adverse mental health outcomes. In addition, our results should be interpreted within the cultural backdrop of the UK, a socially liberal country that affords a degree of social acceptance not present in more socially conservative countries. This has implications for the generalizability of our findings to other contexts and cultures. Therefore, successful interventions supporting sexual minority young people are likely to be most effective when they respond both to the needs of the individual, as well as the wider social and cultural climate.

## 5. Conclusion

We show that identifying as a sexual minority is connected to adverse outcomes in adolescence. In our network, we found a unique and very strong relationship between sexual minority status and depression. Many of the poor outcomes associated with sexual minority status were indirectly related. That is, they are antecedents or consequences of depression and peer/conduct problems. Interventions aimed at reducing poor outcomes among sexual minority adolescents might be most effective if they are targeted towards depression and poor peer/conduct problems. We also identified subgroups of sexual minority adolescents, which highlighted individual differences in adverse outcomes, parental support as a potentially protective factor for advanced depression, and the need for tailored intervention and management.

## Data Availability

Data can be freely accessed through the UK Data Service. Supplementary material is available online [69].
